# Environmental pollutant BDE-47 alters glycan and microRNA signatures of macrophage-derived extracellular vesicles and modulates senescence signaling

**DOI:** 10.1186/s12964-026-02903-2

**Published:** 2026-05-06

**Authors:** Noemi Aloi, Alessia Maria Sampino, Alessia Li Vigni, Ilaria Cosentini, Giuseppa Augello, Fabrizio Chiodo, Gaspare Drago, Silvia Ruggieri, Daniele Romancino, Sabrina Picciotto, Giorgia Adamo, Samuele Raccosta, Mauro Manno, Antonella Bongiovanni, Paolo Colombo, Valeria Longo

**Affiliations:** 1 Institute for Biomedical Research and Innovation, National Research Council of Italy (IRIB-CNR), Via Ugo La Malfa 153, Palermo, 90146 Italy; 2 Department of Biological, Chemical and Pharmaceutical Sciences and Technologies (STEBICEF), University of Palermo, Palermo, Italy; 3 Institute of Biophysics, National Research Council of Italy (IBF-CNR), Palermo, Italy

## Abstract

**Background:**

Extracellular vesicles (EVs) are key mediators of intercellular communication, and their molecular cargo and surface properties can be profoundly influenced by external stimuli. In the context of inflammation, immune cells increase EV release to regulate immunity and metabolism. We previously demonstrated that BDE-47 alters macrophage immune responses by impairing pro-inflammatory cytokine secretion and modulating both the production and microRNA cargo of macrophage-derived sEVs, which in turn can influence the phenotype of naïve macrophages. These observations suggest that pollutant-induced alterations of EVs may represent an important mechanism regulating cell-to-cell communication and immune signaling pathways.

**Methods:**

EV enriched fractions were separated from the conditioned media of BDE-47-treated THP-1 M(LPS) macrophages according to their sedimentation coefficient, and density by differential centrifugation. This procedure enables the enrichment of denser EVs at 10,000 × g (10KEVs), whereas less dense vesicles are predominantly recovered at 100,000 × g (100KEVs). The size, concentration, and surface characteristics of the 10KEVs and 100KEVs were assessed by means of NTA, DLS, Western blotting, and lectin-binding assays, while EV-associated miRNAs were profiled by microarray. Pathway enrichment analysis was conducted to identify key biological pathways altered due to BDE-47 treatment. The downstream effects of different enriched fractions of EVs (100KEVs^DMSO/BDE−47^ and 10KEVs^DMSO/BDE−47^) were evaluated on LNCaP cells by mean of uptake studies, BrdU incorporation and β-galactosidase senescence assays. Furthermore, transcriptional and Western Blot analyses were performed to investigate the expression of genes involved in cell cycle regulation.

**Results:**

Our results show that BDE-47 does not alter EV size and surface canonical markers but profoundly reshapes their molecular identity. Specifically, we observed changes in glycan surface expression and a selective modulation of miRNA sorting in both the 100KEVs and 10KEVs enriched fractions. Bioinformatic analysis revealed a distinct BDE-47–associated EV-miRNA signature linked to the regulation of cell cycle checkpoint pathways. Functional assays performed in LNCaP cells demonstrated that EV fractions derived from BDE-47–treated macrophages (10KEVs^BDE−47/DMSO^ and 100KEVs^BDE−47/DMSO^, respectively) were differentially internalized and exerted distinct biological effects on recipient cells. In particular, the two EVs^BDE−47/DMSO^ enriched fractions differed in their capacity to be up taken by LNCaP cells, modulate cell proliferation, induce cellular senescence, and regulate the expression of key cell cycle inhibitors, including the p16 and p21 genes.

**Conclusions:**

Our findings highlight EVs as central targets and mediators of pollutant-induced cellular effects, unveiling a novel mechanism by which environmental contaminants interfere with EV-mediated communication and influence the behaviour and functions of recipient cells.

**Supplementary Information:**

The online version contains supplementary material available at 10.1186/s12964-026-02903-2.

## Background

 Extracellular vesicles (EVs) are small, membrane-bound particles secreted by cells that play critical roles in various biological functions, particularly in intercellular communication and in both pathological and physiological processes [1]. Extracellular vesicles, released by all cells, transport proteins, lipids, and nucleic acids to mediate cell-to-cell communication throughout tissues and biofluids [2, 3]. EVs can be classified into two major categories: large extracellular vesicles (lEVs), which include microvesicles (MVs) and are enriched in the 10,000 × g pellet following differential centrifugation of conditioned media, and small extracellular vesicles (sEVs), which include exosomes and are enriched in the 100,000 × g pellet. As a key regulatory component of the immune response, macrophages secrete a large set of cytokines and a variety of signals, including EVs [4]. The diversity of macrophage functions depends on their high degree of plasticity, which is a key characteristic of this cell type [5]. In this view, it is challenging to understand the heterogeneity of macrophage derived EVs in response to different stimuli, since EVs are typically analysed in bulk, capturing only the effects of the entire population. More recently, human EVs have also been investigated in exposure science and toxicology for their capability of influencing cell’s functions upon pollutant treatments [6]. Our research group already focused on the effect of an environmental pollutant, the BDE-47 (2,2’,4,4’-Tetrabromodiphenyl ether), on human macrophage activity and the modulation of the expression of microRNA cargo in macrophage-derived EVs stimulated with LPS [7, 8]. BDE-47 is a flame retardant belonging to the PBDE (Polybrominated Diphenyl Ethers) family. It is one of the most common compound used to enhance the fire resistance of materials such as plastics, electronics, furniture, and textiles [9]. Despite PBDEs have been banned from the market, BDE-47 has been detected in the environment, wildlife, and human tissues, raising concerns about its potential health impacts as endocrine disrupting substances [10]. Regarding their effects on the macrophage immune response, we demonstrated that BDE-47 exerts diverse mechanisms of action, including direct immunotoxic effects on macrophages. Specifically, it impairs the secretion of proinflammatory cytokines in M(LPS) macrophages and their miRNA cargo [6–8]. Furthermore, we found that sEVs derived from BDE-47-exposed macrophages can modulate intracellular miRNAs levels and alter the expression of surface markers in naïve resting THP-1 M(0) macrophages, highlighting the immunomodulatory potential of this compound [6]. In this study, we further studied the heterogeneity of EV populations by using differential centrifugation to characterize two enriched EVs fractions referred as 10KEVs^BDE−47/DMSO^ and 100KEVs^BDE−47/DMSO^, defined on their sedimentation properties. To characterize these EV enriched fractions, we performed a comprehensive analysis focusing on five key aspects: (i) their physical properties, including size distribution and morphology; (ii) their surface marker expression to analyse vesicle composition; (iii) their binding to different human carbohydrates-binding proteins; (iv) their miRNA cargo, assessing potential differences in molecular content and functional implications; and (v) their uptake and functional role in modulating the proliferative capacity of prostate cancer LNCaP cells. By elucidating these characteristics, our study provides new insights into how an environmental pollutant such as BDE-47 can shape extracellular vesicle characteristics, highlighting its potential role in the modulation of EV-mediated intercellular communication within the framework of environmental toxicology.

### Methods

### Reagents

The 2,2’,4,4’-tetrabromodiphenyl ether (BDE-47) was purchased from Toronto Research Chemicals (Ontario, Canada) and dissolved in dimethyl sulfoxide (DMSO, Sigma-Aldrich, Milan, Italy, cat. n. D2650) at the concentration of 25 mM. *E. coli* Lipopolysaccharides (LPS, serotype O26:B6) were purchased from Sigma-Aldrich.

### Cell line cultures

The human monocytic leukemia THP-1 cell line (ECACC 88081201) was maintained in culture with RPMI 1640 medium (Gibco Life Technologies, Monza, Italy), supplemented with heat inactivated 10% Fetal Bovine Serum (FBS, Gibco, Life Technologies, Monza, Italy) and 1% antibiotic (5.000 U/mL penicillin, 5.000 µg/mL streptomycin sulphate, both from Sigma-Aldrich, Gibco, Life Technologies). The monocytes were treated for 72 h at 37 °C and 5% CO_2_ with 200 nM phorbol 12-myristate-13-acetate (PMA, Sigma-Aldrich) to obtain the naïve THP-1 M0 macrophage. The THP-1 M(LPS) phenotype was induced by stimulation with 10 ng/mL of LPS for 24 h at 37 °C and 5% CO_2_.

LNCaP cell line (ATCC CRL-1740) was cultured in RPMI 1640 medium supplemented with 10% FBS and 1% antibiotics at 37 °C and 5% CO_2_.

### Enrichment of extracellular vesicles fractions from THP-1 M(LPS) treated with BDE-47

Extracellular vesicles enriched fractions were prepared after incubation of the THP-1 M0 naïve macrophages with 3 µM BDE-47 or 0.0125% DMSO (control vehicle) in RPMI 1640 medium (Gibco Life Technologies, Monza, Italy), supplemented with 10% heat inactivated EV-depleted FBS and 1% antibiotic for 24 h at 37 °C and 5% CO_2_. Thereafter, cells were differentiated in THP-1 M(LPS) phenotype with 10 ng/mL of LPS and incubated for a further 24 h at 37 °C and 5% CO_2_. The supernatants were collected, and EV fractions were separated according to differential ultracentrifugation (dUC) method [11, 12]. For simplicity, the two enriched fractions will hereafter be referred to as 10KEVS^BDE−47^ and 100KEVs^BDE−47^ or 10KEVs^DMSO^ and 100KEVs^DMSO^, respectively, according to their different sedimentation rates and stimulus. Specifically, three independent preparations were used for the subsequent assays. The supernatants were first centrifuged at low speed to remove cells and debris and subsequently centrifuged at 10.000 x g for 30 min at 4 °C to isolate 10KEVs. The 10KEVs were resuspended in a proper volume of microfiltered 1X phosphate buffered saline (PBS) w/o Ca^2+^ and Mg^2+^ and stored at -80 °C until use. Afterwards, 100KEVs were collected from the supernatants into Beckman Coulter polypropylene open top tubes via centrifugation at 118.000 x g for 70 min at 4 °C using a Beckman SW28 rotor. The 100KEVs pellets were washed in 1X PBS w/o Ca^2+^ and Mg^2+^ and centrifuged at 118.000 x g for 70 min at 4 °C and finally, resuspended in 50 µl of microfiltered 1X PBS w/o Ca^2+^ and Mg^2+^.

### Nanoparticle tracking analysis of THP-1 derived 10KEVs and 100KEVs enriched fractions

EVs enriched fractions size distribution and concentration were measured by Nanoparticle Tracking Analysis (NTA) using the NanoSight NS300 (Malvern Panalytical, UK), equipped with a 488 nm laser, a high sensitivity sCMOS camera, a syringe pump, and a second 405 nm laser and a CMOS camera. 10KEVs and 100KEVs samples were diluted in particle-free water (Water, HPLC grade, Sigma-Aldrich, filtered by 20 nm using Whatman Anotop filters) to generate a dilution in which 20–120 particles per frame were tracked and to also obtain a concentration within the recommended measurement range (1–10 × 10^8^ particles/mL). Five experiment videos of 60 s duration were analysed using NTA 3.4 Build 3.4.003 (camera level 15–16). A total of 1500 frames/sample were examined, captured, and analysed by applying instrument-optimized settings with a suitable detection threshold so that the observed particles were marked with red crosses and no more than 5 blue crosses were visible. Further settings, such as blur size and Max Jump Distance, were set to “automatic” and viscosity was set to that of water (0.841–0.844 cP).

### Dynamic light scattering

The vesicle solutions were diluted in 1X PBS and centrifuged at 1000 x g for 10 min at 4 °C to remove any dust particles or aggregates. The supernatant, collected using MilliQ-rinsed pipette tips, was transferred into a quartz cuvette and maintained at 20 °C within a temperature-controlled compartment of a BI200-SM goniometer (Brookhaven Instruments, Nashua, NH, USA). Measurements were conducted using a He-Ne laser source tuned at 633 nm (JDS Uniphase 1136 P) and a single-pixel photon counting detector (Hamamatsu C11202-050, Hamamatsu Photonics Deutschland GmbH, Germany). The intensity autocorrelation function, g_2_(t), was acquired at a scattering angle of 90° using a BI-9000 correlator (Brookhaven Instruments, Nashua, NH, USA) and size distribution P_q_(σ) was calculated by assuming that the diffusion coefficient distribution is shaped as a Schultz distribution, as described in Paterna and coworkers [13]. Two robust parameters were obtained from this analysis: D_z_ (the z-averaged hydrodynamic diameter) and PDI (the polydispersity index, which is an estimate of the distribution width).

### Western blot

Cell lysate, 10KEVs and 100KEVs samples (concentration of the particles used in the assays was 10^12^/mL), from control and treated THP-1 M(LPS) macrophages, were mixed with proper volumes of 5X loading buffer [0.25 M Tris-Cl pH 6.8, 10% sodium dodecyl sulphate (SDS), 50% glycerol, 0.25 M dithiothreitol (DTT), 0.25% bromophenol blue], heated at 100 °C for 5 min and loaded into 10% SDS-PAGE for electrophoretic separation. Then, proteins were blotted onto polyvinylidene fluoride (PVDF) membranes which were blocked with BSA-TBS-T solution [3% powdered with bovine serum albumin in TBST (50 mM Tris HCl pH 8.0, 150 mM NaCl with 0.05% Tween 20)] for 1 h at room temperature, followed by primary antibody incubation overnight at 4 °C. The antibodies used were: anti-Alix (clone 3A9, dil. 1:150 in 3% BSA/1X TBS-T), anti-HSP70 (clone W27 dil. 1:500 in 5% Milk/1X TBS-T), anti-Enolase, anti-βActin (clone A5 and clone AC15, respectively, dil. 1:400 in 3% BSA/1X TBS-T, Santa Cruz Biotechnology, Dallas, Texas, USA), anti-CD63 (rabbit polyclonal, dil.1:500, from Invitrogen), anti-Calnexin (rabbit polyclonal, dil.1:1000, from Novus Biologicals), anti CD81 (clone B11, dil.1:200, from Santa Cruz Biotechnology, USA). The membranes were incubated for 1 h with the horseradish peroxidase-conjugated secondary anti-mouse or anti-rabbit antibodies according to the manufacturer’s instructions (Cell Signaling Technologies Inc., Beverly, MA, USA) and the signals were revealed using Super Signal™ Pierce™ ECL (Thermo Fisher Scientific, Monza, Italy). LNCaP cell lysates were prepared using RIPA buffer (Cell Signaling Technologies). Then, proteins were blotted onto nitrocellulose membranes. After transfer blocking was performed using Odyssey^®^ Blocking Buffer (OBB, LI-COR) diluted in TBS, followed by incubation with primary antibodies anti-β-actin (Sigma-Aldrich), anti-p21 (Cell Signaling Technologies), anti-p53 and anti-p16 (Santa Cruz Biotechnology) diluted in OBB. Detection was carried out using IRDye^®^ 800CW or Alexa Fluor 680-conjugated secondary antibodies (LI-COR; Invitrogen), and membranes were scanned with the Odyssey IR scanner (LI-COR Biosciences, Lincoln, NE, USA). Band intensities were quantified using Odyssey 3.0 software.

### ELISA-based solid-phase assay

A volume of 50 µL from a suspension of vesicles at 2 × 10^7^ particles/mL in PBS (10 mM, pH 7.4), was used to coat NUNC MaxiSorp wells (overnight, 4 °C). After discarding and washing (2 × 150 µL) with calcium and magnesium-containing buffer TSM (20 mM tris(hydroxymethyl)aminomethane (Tris)-HCl, pH 8.0; 150 mM NaCl; 1 mM CaCl_2_; 2 mM MgCl_2_), wells were blocked with 80 µL 1% BSA solution (Sigma-Aldrich, lyophilized powder, ≥ 96%, agarose gel electrophoresis) in TSM at room temperature for 30 min. The blocking solution was discarded and 50 µL of different C-type-lectins including human-Fc Dendritic Cell-Specific Intercellular adhesion molecule-3-Grabbing Non-integrin (DC-SIGN), Macrophage Galactose-type Lectin (MGL) and mannose receptor (MR) at 1 µg/mL were added. After 1 h at room temperature, wells were washed with TSM (2 × 150 µL) and 100 µL of anti-human horseradish peroxidase (0.3 µg/mL, Goat anti-human IgG-HRP from Jackson Immuno) were added. After 30 min, wells were washed with TSM (2 × 150 µL). Finally, 100 µL of a substrate solution (3,3′,5,5′- tetramethylbenzidine, TMB, in citric/acetate buffer, pH 4, and H_2_O_2_) were added. After 10 min at room temperature the reaction was stopped with 50 µL of H_2_SO_4_ (0.8 M) and the optical density (OD) was measured at 450 nm in an ELISA reader. The experiment was performed in duplicate and data were normalized over the signal at 450 nm from the positive controls used for each C-type lectin. Polyacrylamide polymers, functionalized with different glycans were purchased from Lectinity, MW approx. 20 kDa, carbohydrate content around 20% mol.: GalNAcα-OCH_2_CH_2_CH_2_NH_2_ 0030-PA (PAA-Tn, positive control for MGL, 20 µg/mL). Mannans from *Saccharomyces cerevisiae* (positive control for DC-SIGN and MR) was purchased from Sigma-Aldrich and used at 10 µg/mL to coat the ELISA wells. Statistical analysis was performed by means of two-way ANOVA multiple comparison with the Tukey’s multiple comparisons test (alpha 0.05), using GraphPad Prism 10.

### MiRNA purification from 10KEVs and 100KEVs enriched fractions

Purified 10KEVs and 100KEVs from control (10KEVs^DMSO^ and 100KEVs^DMSO^) and treated (10KEVs^BDE− 47^ and 100KEVs^BDE− 47^) THP-1 M(LPS) cells were used for miRNA purification. The same number of 10KEVs and 100KEVs (3 × 10^9^ particles) was used for miRNAs’ extraction. The samples were diluted up to 200 µL with 1X PBS w/o Ca^2+^ and Mg^2+^ and then lysed using 1 mL of QIAzol Lysis Reagent (Qiagen, Milan, Italy). The purification of total RNA enriched in miRNAs was performed according to the miRNeasy Serum/Plasma Kit manufacturer’s protocol (Qiagen). To control the yield, purity, and integrity of samples, 1 µl of a Spike-in mix containing UniSp2 (5’GUACUCGGCUUACGAUCGUAA), UniSp4 (5’GAUGGCAUUCGAUCAGUUCUA) and UniSp5 (5’GAUGCUACGGUCAAUGUCUAAG) miRNAs (Qiagen) was added to the samples before the extraction phases. The miRNA samples were eluted in 15 µl of H_2_O DNAse/RNAse free, and concentrations were evaluated by Nanodrop analysis (NanoDrop™ One/OneC Microvolume UV-Vis Spectrophotometer, Thermo Fisher Scientific, Monza, Italy).

#### MiRNA cargo profiling of 10KEVs and 100KEVs enriched fractions

The cDNA synthesis from 10KEVs and 100KEVs miRNAs was performed using the miRCURY™ LNA RT kit (Qiagen). A mix containing UniSp6 (5’CUAGUCCGAUCUAAGUCUUCGA) and *Caenorhabditis elegans* miR-39-3p (Cel miR39-3p, 5’UCACCGGGUGUAAAUCAGCUUG) exogenous controls was added to reactions according to the manufacturer’s protocol in a final volume of 20 µl. The retro-transcriptions were performed for 60 min at 42 °C. Then, the reverse transcriptase enzyme was inactivated for 5 min at 95 °C. Subsequently, the expression profile of 179 miRNA (reported in Additional Fig. 1) was evaluated using the miRCURY LNA miRNA Focus PCR Panel (panel code: YAHS-106Y, Qiagen). Specifically, the cDNA template was amplified by Real Time analysis (StepOnePlus™ Real Time PCR System, Applied Biosystems, Milan, Italy) and the miRCURY LNA™ SYBR GREEN PCR kit. The Real Time PCR conditions were an initial heat activation step at 95 °C for 2 min and 40 cycles of two-step PCR, denaturation at 95 °C for 10 s, annealing/extension at 56 °C for 1 min. The CT data obtained from control (10KEVs^DMSO^ and 100KEVs^DMSO^) and samples (10KEVs^BDE−47^ and 100KEVs^BDE−47^) extracellular vesicles were analyzed using the Qiagen GeneGlobe miRCURY LNA miRNA PCR Data Analysis software (https://dataanalysis2.qiagen.com/miRCury); the data were normalized using the geNorm “Predefined reference miRNA only” function as references. The miRNAs were considered changed between the two groups if the fold change was < 0.5 (down-regulated miRNA) or the fold change was > 2 (up-regulated miRNA). The miRNAs with a quantification cycle (CT) > 35 were considered undetected.

#### Computational analysis

Pathway enrichment analysis was performed to identify the main biological pathways affected by miRNA modulation in 10KEVs and 100KEVs following BDE-47 treatment. First, the validated gene targets of modulated miRNAs in 10KEVs^BDE−47^ versus 100KEVs^BDE−47^ were identified using the multiMiR R package [14]. Specifically, we retrieved only experimentally validated miRNA-target interactions from three publicly available databases: miRecords, miRTarBase and TarBase. To focus on biologically relevant target genes, we applied a graph-based selection approach. A directed graph was constructed, where miRNAs and their target genes were represented as nodes, and interactions between them as edges. To reduce noise and prioritize the most functionally significant targets, we filtered out genes with a low degree of connectivity, retaining only those interacting with a substantial number of miRNAs (*n* = 4). This step helped refine the analysis by emphasizing genes more likely to play a crucial role in the observed regulatory network. The selected target genes were then subjected to Reactome enrichment analysis using the ReactomePA R package [15]. To streamline the interpretation of enriched pathways, we performed a pairwise similarity analysis based on Jaccard’s similarity index, clustering related pathways and reducing redundancy in the enrichment results.

### In vitro assessment of 10KEVs^DMSO/BDE−47^and 100KEVs^DMSO/BDE−47^uptake by LNCaP cells

The 10KEVs^DMSO/BDE−47^ and 100KEVs^DMSO/BDE−47^ were quantified by means of NTA analysis and 3 × 10^10^ particles were labeled with PKH26 (Sigma–Aldrich). As a matched control for PKH26 labelling, 10KEVs/100KEVs-free microfiltered PBS w/o Ca^2+^ and Mg^2+^ (blank) was identically prepared as the EVs samples. Following ultracentrifugation, cellular supernatants were removed and samples were resuspended in 30 µL of microfiltered PBS w/o Ca^2+^ and Mg^2+^. Samples were then resuspended in an equal volume of diluent C with 8 µM PKH26. After incubation at 37 °C for 15 min of staining time, samples were ultracentrifuged at 118.000 x g for 70 min at 4 °C using a Beckman SW28 rotor to remove excess dye. Blank, 10KEVs^DMSO/BDE−47^ and 100KEVs^DMSO/BDE−47^ were resuspended in 30 µL microfiltered PBS w/o Ca^2+^ and Mg^2+^.

LNCaP cells were seeded in 16 well Chamber Slide w/Cover Glass Slide Sterile (Thermo Scientific Nunc) at a density of 10.000 cells/well. After 24 h from culturing cells were treated with PKH26-labelled EVs or blank for 48 h at 37 °C and 5% CO_2_ using an Olympus fluorescent microscope (Olimpus Evident iX3).

### LNCaP cell proliferation assay

Cell proliferation was assessed by measuring bromodeoxyuridine (BrdU) incorporation into DNA using a colorimetric immunoassay (Roche Diagnostics GmbH, Mannheim, Germany), following the manufacturer’s instructions. Briefly, LNCaP cells were seeded at the concentration of 5.000 cells/well in 96-well plate to test the effect of EVs on cellular proliferation. Cells were left to adhere for 24 h. Subsequently, they were incubated with 10/100KEVs^DMSO/BDE−47^ at a concentration of 10^12^/mL for 72 h. BrdU was added 16 h before the end of treatments. Values were expressed as means of OD ± SD of four separate experiments, each performed in triplicate. A statistical analysis was performed using a one-way ANOVA and Bonferroni’s post-hoc analysis test.

### Senescence-associated β-galactosidase assays

LNCaP cells (2 × 10^4^) were grown on an 8-well chamber slide. At time 0, the medium was replaced with a fresh complete medium with the 10KEVs^DMSO/BDE−47^ and 100KEVs^DMSO/BDE−47^ enriched fractions at a concentration of 10^12^/mL for 6 days. After treatments, the cells were fixed and stained for β-galactosidase activity, using a Senescence Cell Staining kit, following the manufacturer’s instructions (Sigma‐Aldrich). The percentage of senescence‐associate β‐gal positive cells was determined by counting the number of blue cells within a sample, using Olympus CKX53 (Olympus Corporation, Tokyo, Japan) microscope with an X20 lens. Ten random fields were photographed for each condition, and the percentage of SA‐β‐gal‐positive cells was calculated. Data are presented as the mean percentage ± standard deviation (SD). Statistical significance between groups was determined using a two-tailed unpaired Student’s t-test.

### RNA isolation, retrotranscription and Digital PCR analysis

Total RNA from LNCaP cells treated with the 10KEVs^DMSO/BDE−47^ and 100KEVs^DMSO/BDE−47^ was isolated according to RNeasy mini kit protocol (Qiagen). 1 µg of each RNA template was retro-transcribed using the QuantiTect Reverse Transcription Kit (Qiagen). The cDNA was diluted 1:500 and the expression of p16 (or CDKN2A, NM_000077) and p21 (or CDKN1A, NM_000389) genes was evaluated by Digital PCR technology using the QIAcuity One System, the EVA Green detection method and the Quantitect primer assays (Qiagen). Specifically, 40 µl of reaction mixtures were prepared in 96 well plates according to the QIAcuity EG PCR Kit manufacturer’s protocol (Qiagen) and subsequently dispensed into the Qiacuity™ Nanoplate 26k 24-well (Qiagen). Positive (the human β-actin gene, NM_001101) and negative (no template reactions) controls were included in each experiment (*n* = 4 independent replication). The cycling profile consisted of a denaturation step at 95 °C for 2 min, 40 cycles at 95 °C for 15 s, 56 °C for 15 s and 72 °C for 15 s, followed by 40 °C for 5 min. The imaging step was performed selecting the green channel and setting the exposure duration at 300 ms and Gain = 3. Data were analyzed by means of QIAcuity Suite Software version 2.5.0.1 (Qiagen) and the absolute quantities were reported as copy number/µl. Any values above 0 copies/µl were considered as positive. The statistical analysis was performed using one-way ANOVA.

## Results

### Biophysical characterization of 10KEVsDMSO/BDE−47 and 100KEVsDMSO/BDE−47 enriched fractions derived from BDE-47 treated THP-1 M (LPS) macrophages

**Fig. 1:**
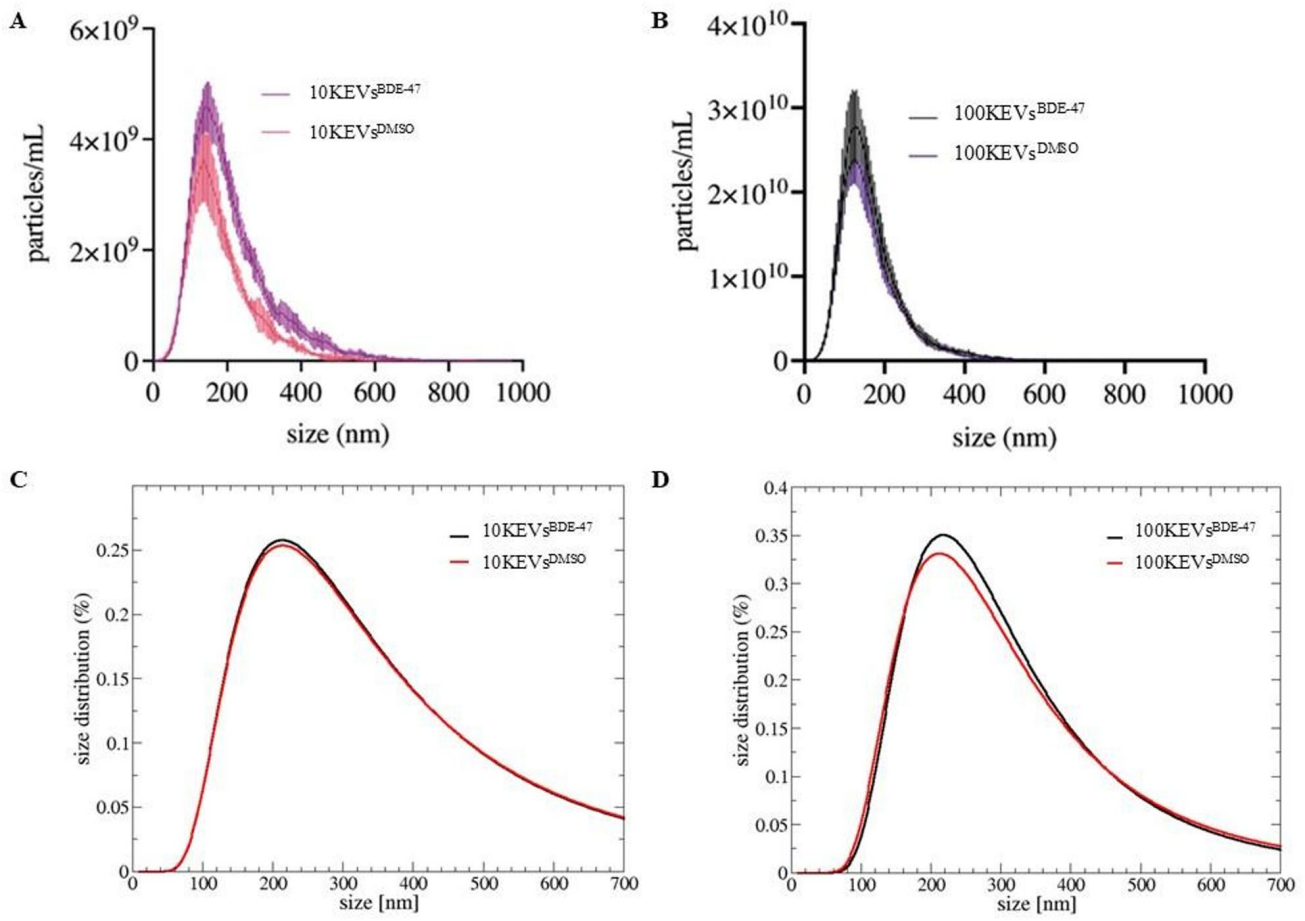
Characterization of EVs enriched fractions derived from THP-1 M(LPS) cells. Representative size distribution of 10KEVs and 100KEVs (panel **A** and **B**) derived respectively from control (DMSO) and BDE-47 treated cells, by NTA. 10KEVs and 100KEVs size distributions were evaluated also by DLS (panel **C** and **D**)

The conditioned media from three independent preparations of THP-1 M(LPS) macrophages treated with BDE-47 or DMSO (control) were collected. The 10KEVs and 100KEVs from each preparation (100KEVs^BDE−47^/10KEVs^BDE−47^ and 100KEVs^DMSO^/10KEVs^DMSO^, respectively) were isolated according to the dUC method. To assess whether exposure to the environmental pollutant BDE-47 influences the size distribution and particle concentration of EVs, we performed Nanoparticle Tracking Analysis on the EVs subtypes isolated from treated and untreated cells. The size distribution profiles of 100KEVs and 10KEVs revealed no significant or only slight differences between control (DMSO) and BDE-47-treated samples with overlapping curves indicating comparable particle size distribution profile (Fig. 1A and B). These findings were also supported by Dynamic Light Scattering analyses (Fig. 1C and D). In Tables 1 and 2, we have summarized concentration values and the size distributions of EVs subtypes obtained by NTA and DLS assays.

**Table 1:** Concentrations and size distributions of EVs enriched fractions

EV subtypes	Concentration (10^9^ particles/mL)	Size (nm)
10KEVs ^DMSO^	160 ± 14	114 ± 12
10KEVs^BDE−47^	125 ± 40	114 ± 22
100KEVs^DMSO^	300 ± 170	140 ± 15
100KEVs^BDE−47^	590 ± 160	125 ± 22

**Table 2:** EVs enriched fractions parameters derived by Dynamic Light Scattering analyses

EVs subtypes	D_z_ (nm)	PDI
10KEVs^DMSO^	265 ± 5	0,22
10KEVs^BDE−47^	265 ± 5	0,25
100KEVs^DMSO^	292 ± 5	0,37
100KEVs^BDE−47^	294 ± 5	0,38

### Evaluation of EVs Markers

**Fig. 2:**
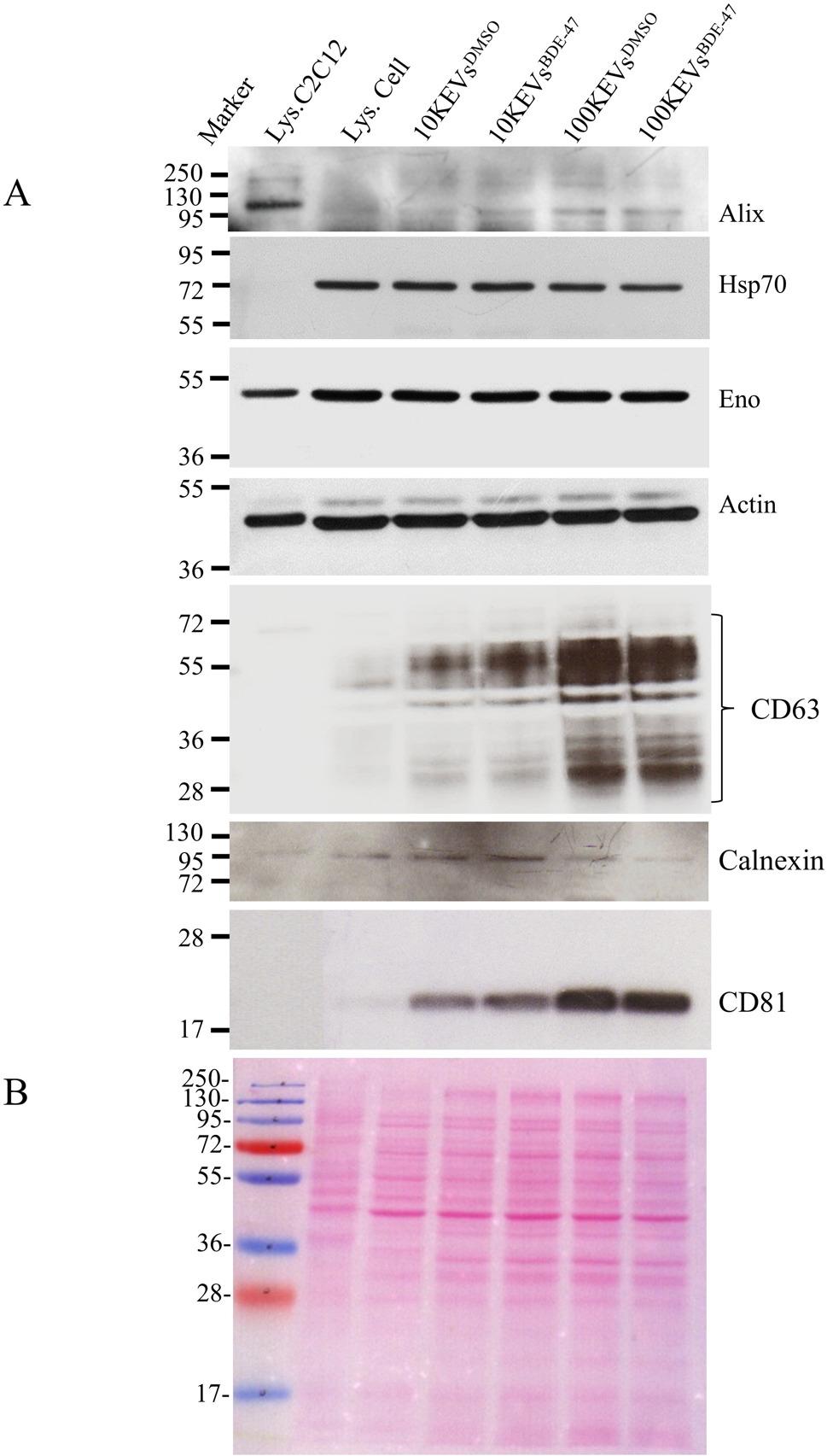
Immunoblot analyses of 100KEVs and 10KEVs biomarkers. Analyses of Alix, β-actin, enolase (Eno), and Hsp70, CD63, CD81 and Calnexin in THP-1 M(LPS) derived of 10KEVs^DMSO/BDE−47^ and/or 100KEVs^DMSO/BDE−47^ enriched fractions. Equal amounts of total proteins were loaded for THP-1 M(LPS) cell lysates and EVs samples (10 µg/lane). Lysate from C2C12 cells (8 µg) was used as positive control (**A**). Ponceau staining is shown as a control of the total protein loaded per lane (**B**)

The 10KEVs and 100KEVs enriched fractions were analysed by Western blot to assess the expression of specific markers associated with each EV fraction. This approach allowed us to evaluate the identity and purity of the isolated EV fractions based on established molecular signatures. Specifically, we assessed the expression of Enolase-1 (Eno-1), Hsp70, Alix, CD63, CD81 and Calnexin. Our findings are consistent with the MISEV 2018 and MISEV 2024 Guidelines [11, 12]. Indeed, we found a higher expression of CD63 and CD81 in 100KEVs subtypes (100KEVs^DMSO^/100KEVs^BDE−47^) compared to 10KEVs, while the Calnexin showed greater expression in the 10KEVs subpopulations (10KEVs^DMSO^/10KEVs^BDE−47^). No significant differences were found when comparing BDE-47-derived EV fractions to control EVs (10KEVs^DMSO^ versus 10KEVs^BDE−47^ and 100KEVs^DMSO^ versus 100KEVs^BDE−47^, respectively). These results suggest that BDE-47 does not interfere with the expression of specific EVs markers. The immunoblot data are shown in Fig. 2 panels A and B.

### Evaluation of BDE-47’s effects on 10KEVs and 100KEVs binding to human lectins

**Fig. 3:**
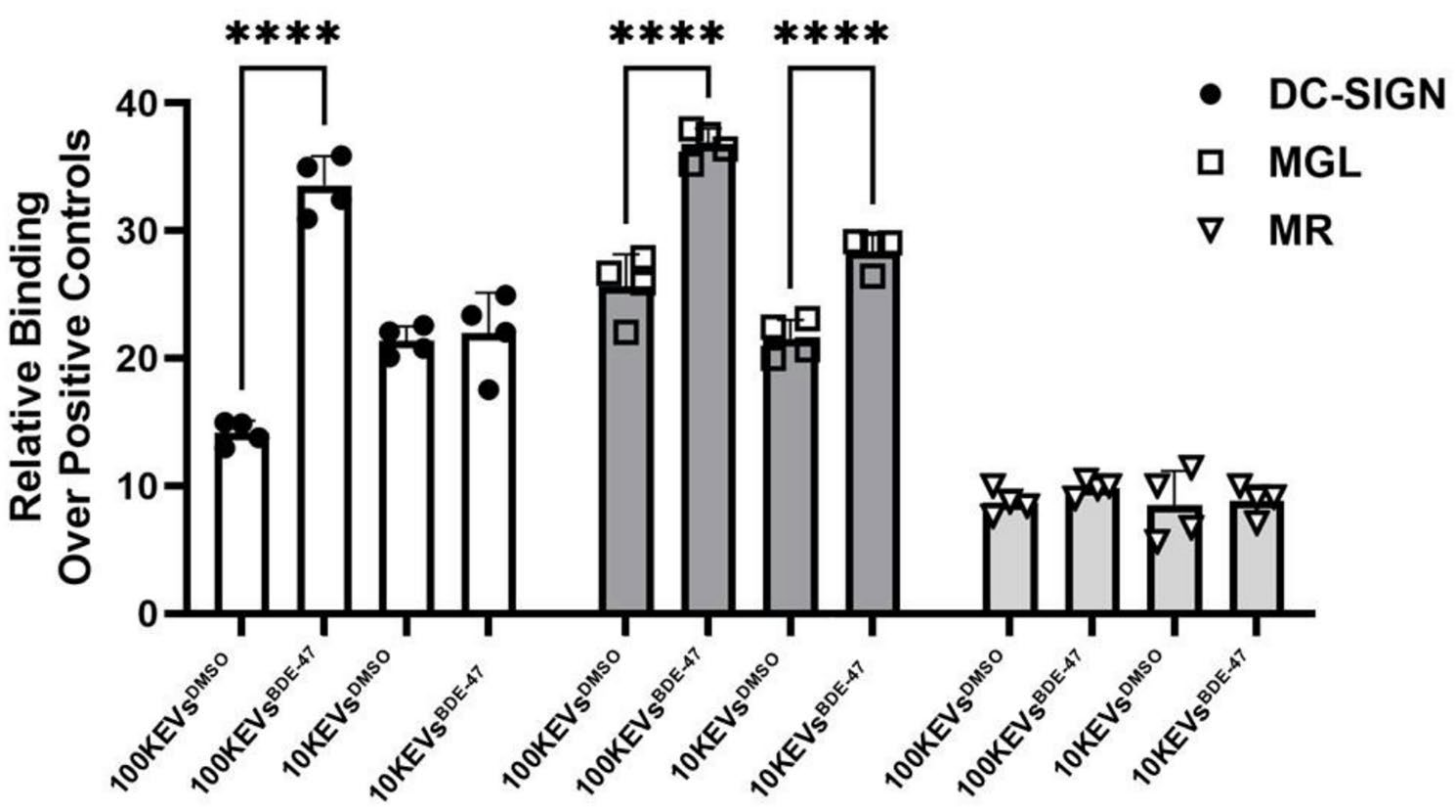
Binding of 100KEVs and 10KEVs from BDE-47 or DMSO treated THP-1 macrophages to human C-type lectins. Data were normalized over signal from the lectins-positive controls (glycosylated polyacrylamide polymers or Mannans, see also Additional Fig. 2). Error bars indicate standard deviations. Two-way ANOVA multiple comparison was performed with the Tukey’s multiple comparisons test (alpha 0.05), using GraphPad Prism 10. (*: *p* < 0.05, **: *p* < 0.01, ****: *p* < 0.0001)

To study the 10KEVs^DMSO/BDE−47^ and/or 100KEVs^DMSO/BDE−47^ binding to human lectins, we employed an ELISA-based solid-phase assay to evaluate EV interactions with the C-type lectins DC-SIGN, Langerin, MR, and MGL. As shown in Fig. 3, EVs exhibited significant binding to DC-SIGN and MGL, consistent with the presence of high-mannose, fucosylated, or α-GalNAc residues on the N or O-glycan structures on their surface. Interestingly, the MR, which preferentially binds terminal mannoses, shows non-detectable interactions in the tested experimental conditions. These data suggested that in the 10KEVs^BDE−47^ there is a higher presence of C-type lectins-specific glycan structures compared to both the 10KEVs^DMSO^ and the 100KEVs^DMSO^/^BDE−47^ groups. Furthermore, we observed a higher interaction between 100KEVs^BDE−47^ and MGL than 100KEVs^DMSO^. These findings suggest that EVs may utilize C-type lectin recognition to mediate immune modulation, and that BDE-47 may influence the expression of lectin-specific glycan structures on the surfaces of EV enriched fractions.

### BDE-47 modulates miRNA sorting into macrophage-derived 100KEVs and 10KEVs enriched fractions

**Fig. 4:**
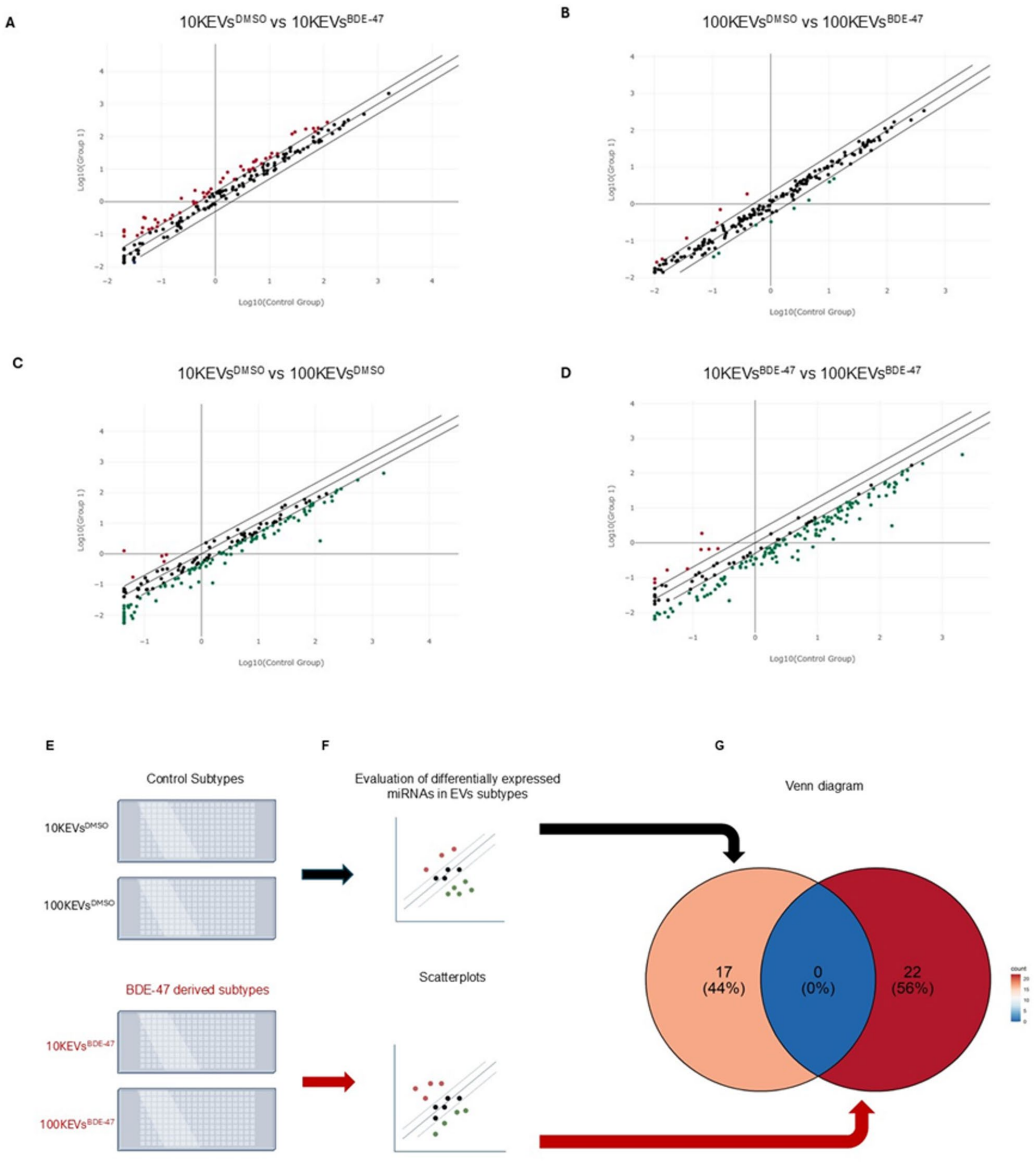
Microarray analysis and workflow for identifying differentially expressed miRNAs between 10 K and 100 K EVs from THP-1 M (LPS) after BDE-47 treatment. The scatterplots show the differential expressed miRNAs comparing 10KEVs^DMSO^ vs. 10KEVs^BDE−47^ (**A**), 100KEVs^DMSO^ vs. 100KEVs^BDE−47^ (**B**), 10KEVs^DMSO^ vs. 100KEVs^DMSO^ (**C**) and 10KEVs^BDE−47^ vs. 100KEVs^BDE−47^ (**D**). Panels (**E**–**F**) illustrate the schematic workflow used to identify differentially expressed miRNAs between 10KEVs and 100KEVs-enriched fraction in control (10KEVs^DMSO^ vs100KEVs^DMSO^ and BDE-47-treated conditions (10KEVs^BDE−47^vs. 100KEVs^BDE−47^). The Venn diagram (**G**) shows the number of significantly modulated miRNAs in DMSO-derived fractions (orange circle, *n* = 17) and BDE-47-derived fractions (red circle, *n* = 22). No common miRNAs were identified between the two fraction groups (blue intersection area, *n* = 0). Figure partially created with BioRender

In a previous study, we demonstrated that the flame-retardant BDE-47 can modulate the expression of miRNAs cargo in macrophage derived 100KEVs [6]. Here, we have expanded our analysis to focus on BDE-47 ability to rewire miRNA sorting between different EVs enriched fractions. In these perspectives, the 10KEVs and 100KEVs fractions from conditioned culture media collected from control (10KEVs^DMSO^ and 100KEVs^DMSO^) or BDE-47 (10KEVs^BDE−47^ and 100KEVs^BDE−47^) treated THP-1 M(LPS) macrophage were profiled for their miRNAs detecting the level of expression of 179 miRNAs by means of microarray assays. First, we examined whether BDE-47 exposure modulates the miRNA cargo in the 10KEVs– or 100KEVs–enriched fractions by comparing miRNA expression profiles between the 10KEVs^DMSO^ and 10KEVs^BDE−47^ groups and, likewise, between the 100KEVs^DMSO^ and 100KEVs^BDE−47^ groups. Data confirmed the ability of BDE-47 to reprogram the miRNA profile in both EV-enriched fractions, as illustrated in the scatterplots reported in Figs. 4A and B. The significantly modulated miRNAs and the corresponding fold change values in the different EV-enriched fractions ​​are reported in Tables 3 and 4 respectively.

**Table 3:** **MiRNAs differentially expressed in 10KEVs** ^**BDE−47**^ **compared to 10KEVs** ^**DMSO**^ **and corresponding fold change values**

miRNA ID	Fold Change
hsa-miR-106a-5p	2.06
hsa-miR-20a-5p	2.35
hsa-miR-17-5p	2.32
hsa-miR-101-3p	3.82
hsa-let-7 g-5p	2.49
hsa-miR-32-5p	6.91
hsa-miR-140-5p	2.97

**Table 4:** MiRNAs differentially expressed in 100KEVs^BDE−47^ compared to 100KEVs^DMSO^ and corresponding fold change values

miRNA ID	Fold Change
hsa-miR-26a-5p	0.39
hsa-miR-186-5p	0.32
hsa-miR-26b-5p	0.30
hsa-miR-320b	0.40
hsa-miR-126-5p	0.48
hsa-miR-195-5p	0.36
hsa-miR-320d	0.28
hsa-miR-150-5p	4.74
hsa-miR-133a-3p	0.35
hsa-miR-136-5p	2.37
hsa-miR-2110	2.58
hsa-miR-144-5p	2.32
hsa-miR-7-1-3p	3.27
hsa-miR-151a-3p	5.15

To determine whether BDE-47 influences the miRNA signature of the 10KEVs- and 100KEV-enriched fractions, we extended our analysis by comparing the miRNA cargo of these two fractions, following the schematic workflow shown in Fig. 4 (panels E and F). The comparative analysis of differentially expressed miRNAs between 10KEVs^DMSO^ and 100KEVs^DMSO^ and 10KEVs^BDE−47^ vs. 100KEVs^BDE−47^ (scatterplots in Fig. 4C and D), revealed distinct miRNA profiles in each condition. Specifically, 10KEVs^DMSO^ and 100KEVs^DMSO^ differed in their miRNA cargo, with 17 significantly modulated miRNAs. In contrast, comparison of 10KEVs^BDE−47^ vs. 100KEVs^BDE−47^ fractions showed modulation of 22 miRNAs. We summarized the modulated miRNAs and the corresponding fold changes value in Tables 5 and 6 respectively. In addition, the Venn diagram shown in Fig. 4 panel G highlights the specific contribution of BDE-47 to miRNA loading by comparing the subgroups 10KEVs^DMSO^ vs. 100KEVs^DMSO^ and 10KEVs^BDE−47^ vs. 100KEVs^BDE−47^ by means of orange and red circles. Notably, the two clusters do not share any commonly modulated miRNAs within the selected panel (blue area).

**Table 5:** Differential expressed miRNAs between 10KEVs^DMSO^ and 100KEVs^DMSO^

*N*.	miRNA ID	Fold Change
1	hsa-miR-451a	28.75
2	hsa-miR-200a-3p	0.20
3	hsa-miR-485-3p	0.13
4	hsa-miR-154-5p	0.13
5	hsa-miR-382-5p	0.38
6	hsa-miR-532-5p	0.50
7	hsa-miR-497-5p	0.19
8	hsa-miR-136-5p	0.17
9	hsa-miR-208a-3p	0.13
10	hsa-miR-629-5p	0.29
11	hsa-miR-22-5p	0.29
12	hsa-miR-144-5p	0.26
13	hsa-miR-92a-3p	0.47
14	hsa-miR-483-5p	0.13
15	hsa-miR-151a-3p	0.07
16	hsa-miR-136-3p	0.13
17	hsa-miR-543	0.16

**Table 6:** Differential expressed miRNAs between 10KEVs^BDE−47^ and 100KEVs^BDE−47^

*N*.	miRNA ID	Fold Change
1	hsa-miR-486-5p	0.18
2	hsa-miR-125b-5p	0.27
3	hsa-miR-150-5p	0.21
4	hsa-miR-221-3p	0.33
5	hsa-let-7f-5p	3.62
6	hsa-miR-27b-3p	0.22
7	**hsa-m-5piR-106a**	**0.37**
8	hsa-let-7a-5p	0.17
9	hsa-let-7b-3p	0.29
10	hsa-let-7b-5p	0.19
11	hsa-miR-30e-5p	0.24
12	hsa-miR-148b-3p	0.27
13	**hsa-miR-20a-5p**	**0.31**
14	**hsa-miR-17-5p**	**0.40**
15	hsa-miR-101-3p	0.45
16	hsa-miR-23a-3p	0.41
17	hsa-miR-223-3p	2.68
18	hsa-let-7d-3p	0.25
19	hsa-miR-192-5p	13.35
20	**hsa-miR-18a-5p**	**0.37**
21	hsa-miR-140-5p	0.34
22	hsa-miR-584-5p	0.22

### Pathway enrichment analysis

**Fig. 5:**
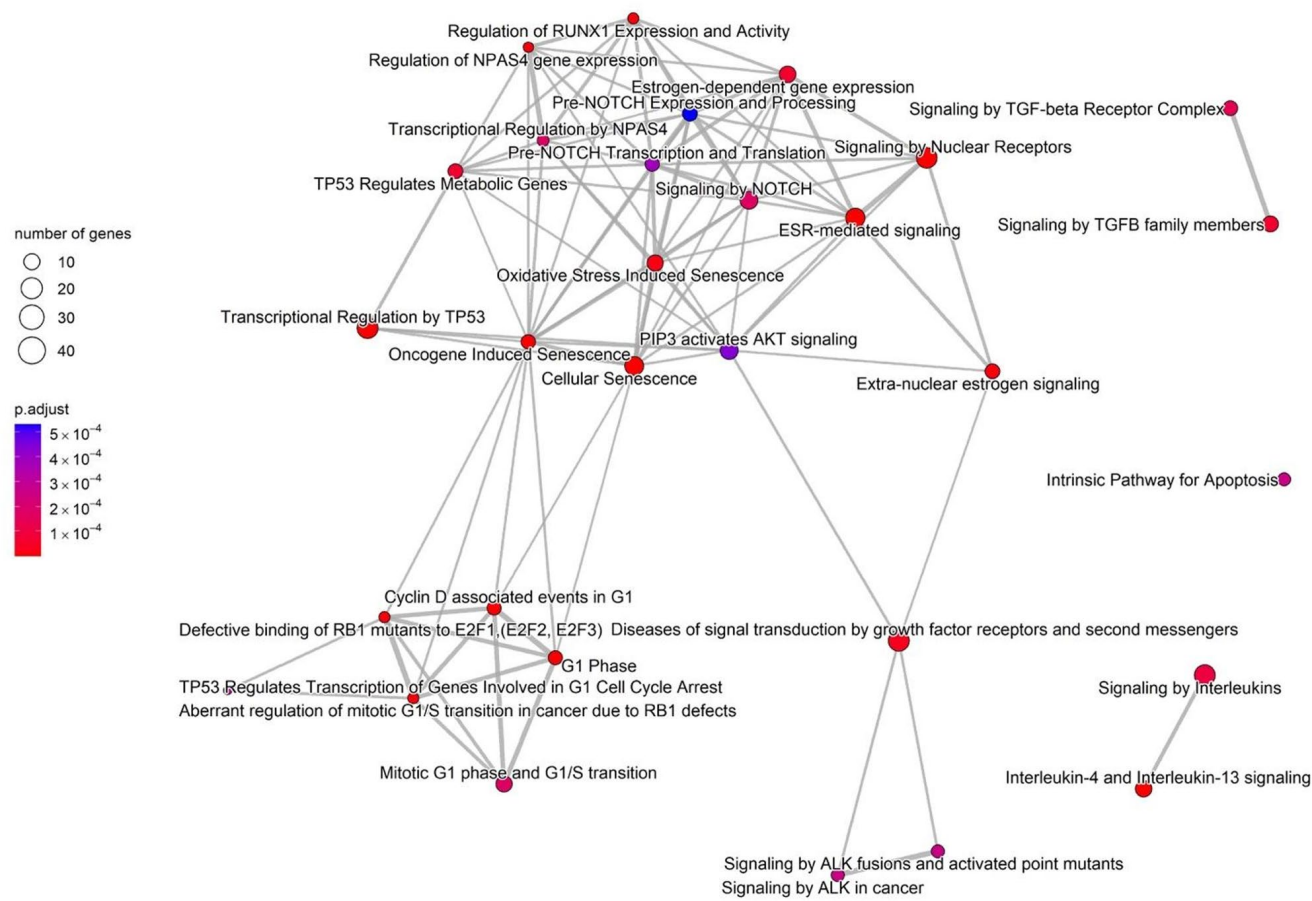
Pathway enrichment analysis. Network diagram illustrates the connections between enriched pathways, where nodes represent biological Reactome pathways and edges indicate shared genes between them

**Fig. 6:**
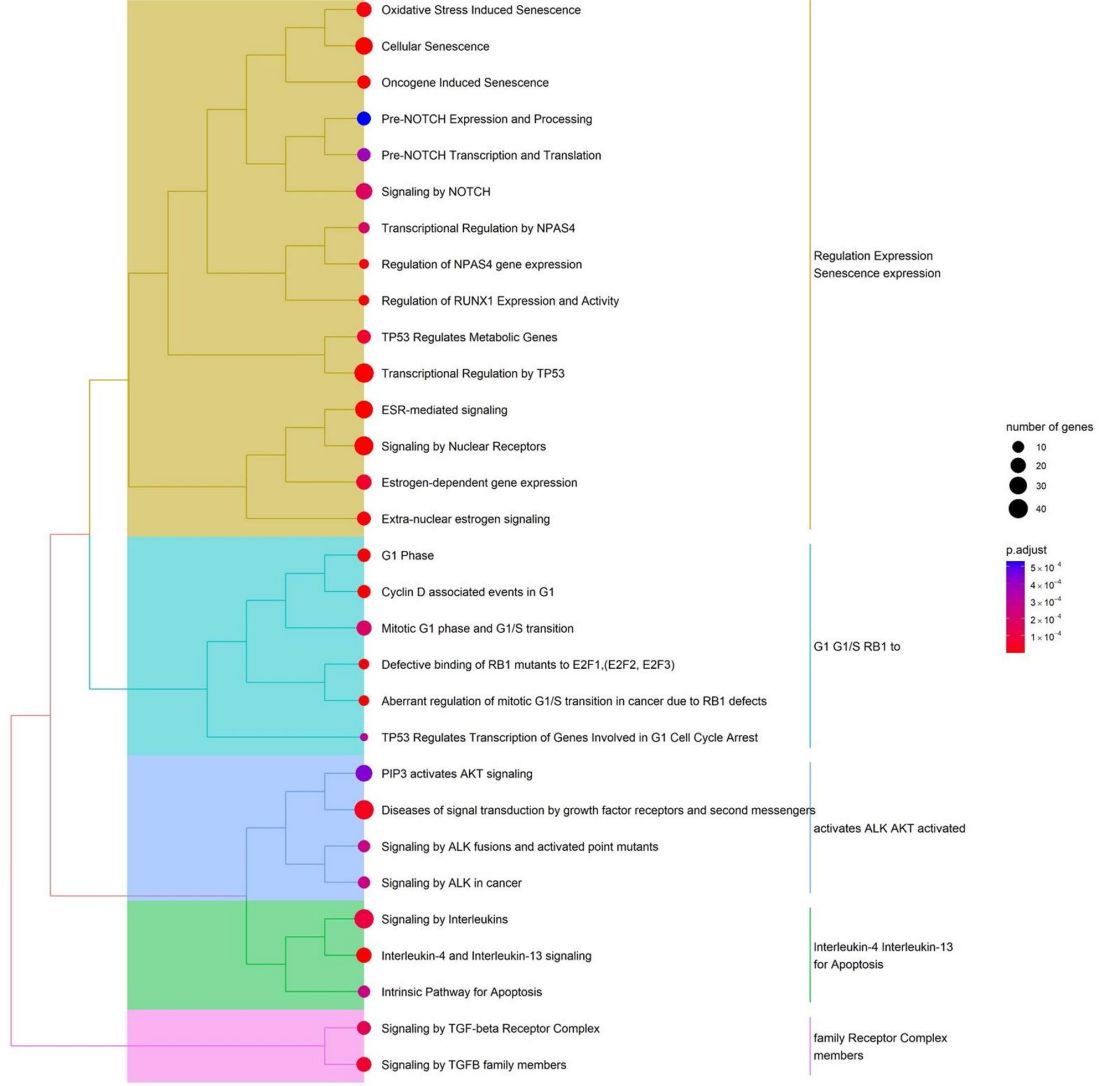
Pathway enrichment analysis using the Reactome database. Three plots illustrate the relationship between various biological pathways grouped by hierarchical clustering of enriched terms. The size of nodes indicates the number of miRNA-target genes associated with each pathway. The nodes colour gradient represents the adjusted p-value, with blue indicating higher values of 5 × 10^− 4^ and red lower values of 5 × 10^− 4^

Microarray analysis was used to investigate global transcriptional changes and their functional implications. The network diagram in Fig. 5 illustrates the connections between enriched pathways, where nodes represent biological Reactome pathways and edges indicate shared genes between them. The size of each node corresponds to the number of genes involved, while the colour scale represents the adjusted p-value (p. adjust), with darker colours indicating higher statistical significance. The pathways are organized into functional groups, with key clusters including senescence-associated processes, cell cycle regulation, and signalling cascades such as NOTCH, AKT, and ALK. Figure 6 presents a hierarchical clustering dendrogram of the same enriched pathways, grouping functionally related pathways based on gene overlap. The clustering reveals distinct modules corresponding to major biological themes, such as senescence regulation, G1/S cell cycle transition, AKT activation, and interleukins signalling. The color-coded background highlights key pathway categories, further emphasizing the functional relationships among the enriched pathways. Overall, the enrichment analysis indicates a strong association of the dataset with cellular senescence, transcriptional regulation, and oncogenic signalling (Fig. 6). It is relevant to note that several members of the miR17 and miR18 clusters (reported in bold in Table 6) have been modulated (i.e. hsa-miR-106a-5p, hsa-miR-20a-5p, and hsa-miR-17-5p). These microRNAs are key regulators of pro-inflammatory responses and have been implicated in multiple cancers, including thyroid, prostate, and colorectal cancers, as well as glioma and melanoma [16–18]. These miRNAs converge on the p53–p21/p16–Rb checkpoints and SASP (Senescence-Associated Secretory Phenotype) -linked cytokine networks, thereby aligning mechanistically with the pathway enrichment results (cellular senescence, transcriptional regulation, oncogenicsignalling).

### Uptake studies of 10KEVs^DMSO/BDE−47^and 100KEVs^DMSO/BDE−47^enriched fractions in LNCaP cells

**Fig. 7:**
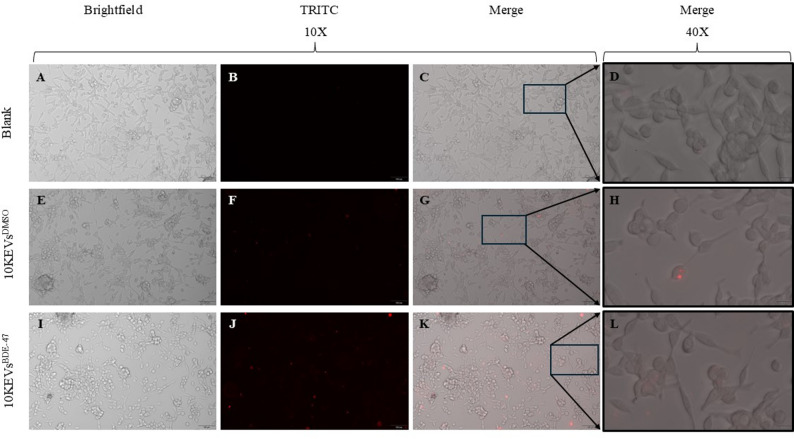
10KEVs^BDE−47/DMSO^ uptake studies.Representative fluorescence microscopy images illustrating the cellular uptake of PKH26-labeled Blank, 10KEVs^DMSO^, and 100KEVs^BDE−47^ in LNCaP cells. Panels **A**–**C**, **E**–**G**, and **I**–**K** show the brightfield, TRITC, and merged channels at 10X magnification. Panels **D**, **H**, and **L** present merged images of representative fields acquired at 40X magnification

**Fig. 8:**
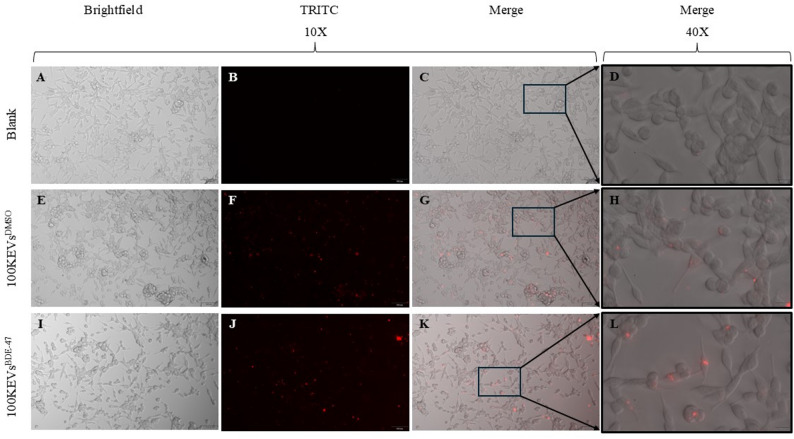
100KEVs^BDE-47/DMSO^ uptake studies. Representative fluorescence microscopy images illustrating the cellular uptake of PKH26-labeled Blank, 100KEVs^DMSO^, and 100KEVs^BDE-47^ in LNCaP cells. Panels **A**–**C**, **E**–**G**, and **I**–**K** show the brightfield, TRITC, and merged channels at 10X magnification. Panels **D**, **H**, and **L** present merged images of representative fields acquired at 40X magnification

In silico analyses have suggested a potential involvement of the EV-enriched fractions in regulating the molecular mechanisms underlying cellular senescence. In this context, we investigated the functional activity of both 10KEVs^DMSO/BDE−47^ and 100KEVs^DMSO/BDE−47^ fractions in LNCaP cells performing fluorescence microscopy analysis. Therefore, the LNCaP cells were treated for 48 h with PKH26-labeled 10KEVs^DMSO/BDE−47^ and 100KEVs^DMSO/BDE−47^. Microscopy analyses, reported in Fig. 7, showed that cells incubated either with 10KEVs^DMSO^ (panels E-F-G, 10X magnification and H, 40X magnification) or with 10KEVs^BDE−47^ (panels I-J-K, 10X magnification and L, 40X magnification) displayed a more diffuse and less structured fluorescence signal within the cytoplasmic area and internalization was not observed. In contrast, as shown in Fig. 8, LNCaP cells incubated with PKH26-labeled 100KEVs^DMSO^ (panels E-F-G, 10X magnification and H, 40X magnification) exhibited a detectable intracellular red fluorescence signal. LNCaP cells treated with 100KEVs^BDE−47^ (panels I-J-K, 10X magnification and L, 40X magnification) showed a more intense and widespread fluorescence signal compared to those treated with 100KEVs^DMSO^. The fluorescence signal appeared distributed within the cytoplasmic compartment, consistent with vesicle uptake. No red fluorescence signal was detected in the blank condition (Figs. 7 and 8, panels A–B–C at 10× magnification and panel D at 40× magnification), confirming the absence of nonspecific staining or autofluorescence. Overall, these results suggest efficient internalization of 100KEVs^DMSO/BDE−47^ by LNCaP cells with respect to 10KEVs^DMSO/BDE−47^. Notably, LNCaP cells treated for 48 h with 100KEVs^BDE−47^ appeared less confluent than the same cells treated with 100KEVs^DMSO^.

### Effects of 10KEVs and 100KEVs enriched fractions on LNCaP cells: analyses of cell proliferation

**Fig. 9:**
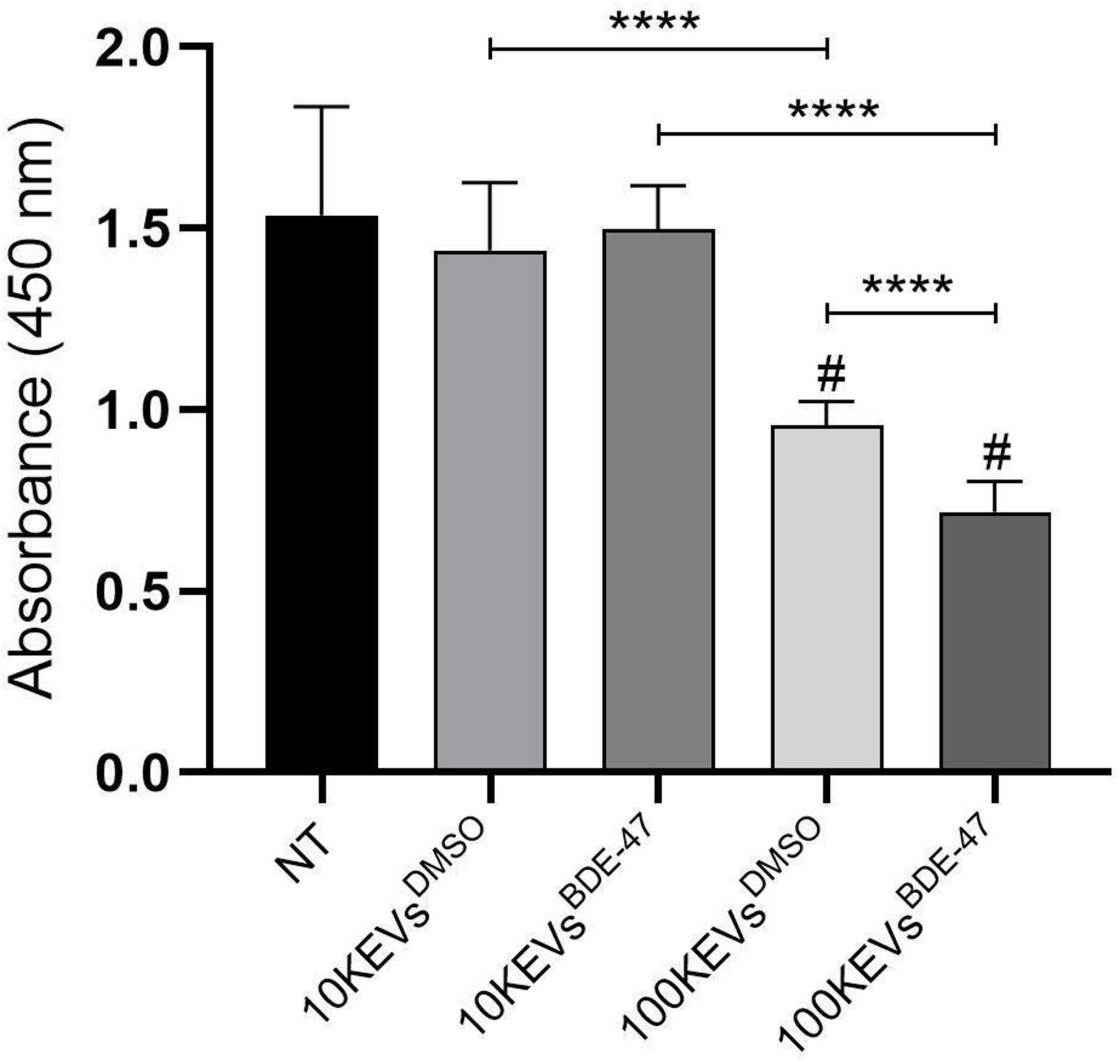
Cell proliferation assay. DNA synthesis was measured by BrdU incorporation into DNA. LNCaP cells were treated with 10KEVs^DMSO/BDE−47^ or 100KEVs^DMSO/BDE−47^ at a concentration of 10^12^/mL for 72 h. NT indicates untreated cells. Data are expressed as absorbance at 450 nm of the means ± SD of four separate experiments, each of which was performed in triplicate(****p < 0.001)

Based on insights from our bioinformatic analysis, a BrdU incorporation assay was conducted to evaluate the potential functional role of EVs in modulating the proliferative capacity of heterologous cell lines, specifically LNCaP cells. To this end, human prostate cancer cell line LNCaP were treated for 72 h with either 10KEVs or 100KEVs derived from BDE-47- or DMSO-treated THP-1 M(LPS) cells. Figure 9 shows that both 100KEVs^BDE−47^ and 100KEVs^DMSO^ significantly reduce LNCaP cell proliferation compared to their respective 10 K counterparts. Notably, 100KEVs^BDE−47^ exert a more pronounced antiproliferative effect than 100KEVs^DMSO^, suggesting that BDE-47 exposure may enhance the functional capacity of 100KEVs to suppress cell proliferation. Untreated cells display a proliferative rate comparable to the 10KEVs^DMSO/BDE−47^.

### Effects of 10KEVs^BDE−47/DMSO^ or 100KEVs^BDE−47/DMSO^enriched fractionson LNCaP cells: analyses of senescence-markers

**Fig. 10:**
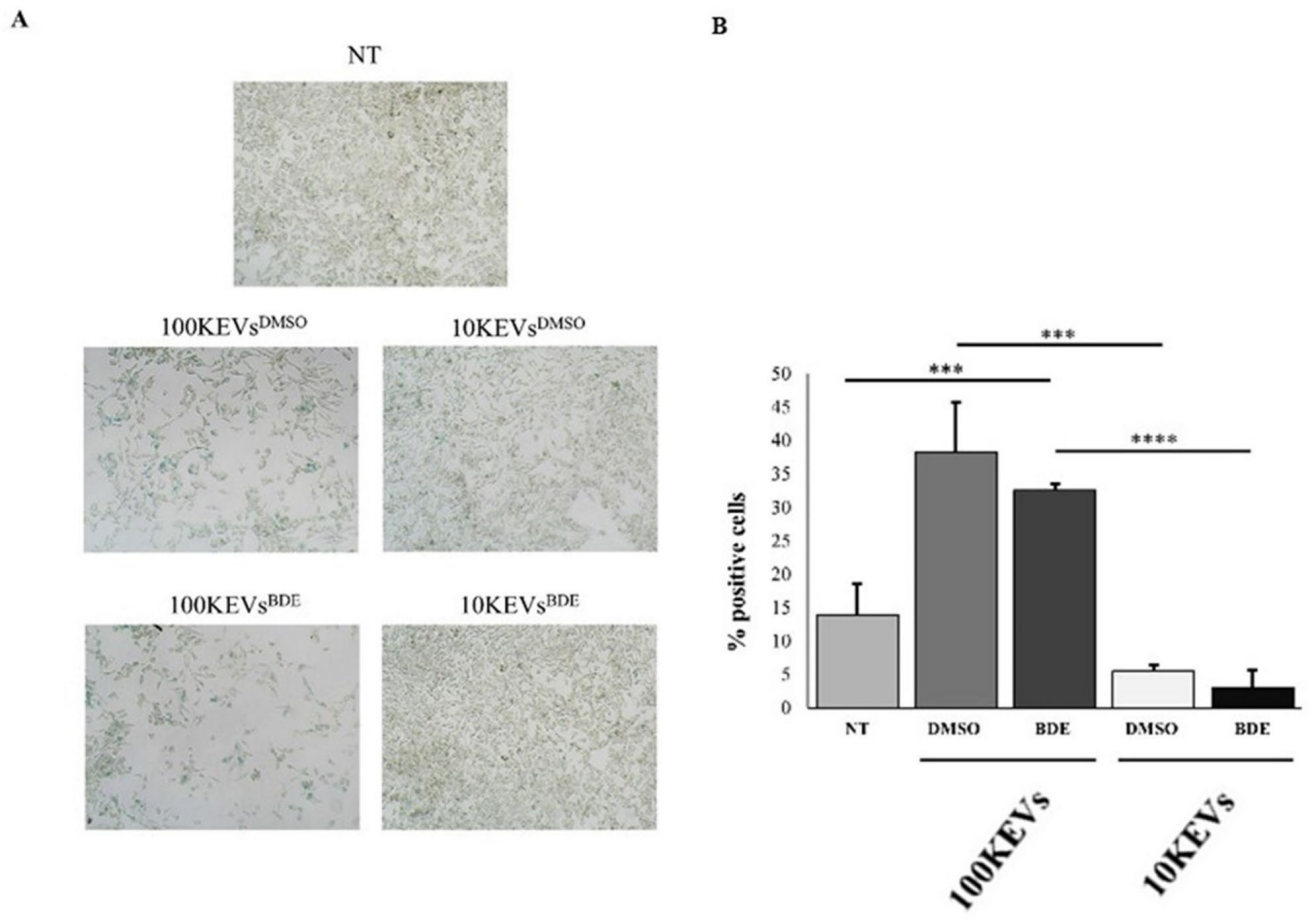
Cell morphology and senescence-associated β-galactosidase (SA-β-Gal) activity in control LNCaP cells (NT) and treated with 10KEVs/100KEVs^DMSO/BDE− 47^ at a concentration of 10^12^/mL for 6 days (**A**). The graph displays the percentages of SA-β-Gal-positive cells (**B**, ***p < 0.005, ****p < 0.001 respectively)

To further explore the underlying mechanisms contributing to the above reported antiproliferative effect and to confirm the results of our bioinformatic analysis, we next investigated whether these EVs enriched fractions might be involved in the induction of cellular senescence. LNCaP cells were exposed to 10KEVs^BDE−47/DMSO^ or 100KEVs^BDE−47/DMSO^ for six days, followed by evaluation using the senescence-associated β-galactosidase assay. Microscopic analysis and quantification of β-galactosidase-positive cells revealed a marked reduction in cell number following treatment with 100KEVs confirming our proliferation assay. Additionally, the remaining cells exhibited a predominant blue coloration, indicative of senescence-associated β-galactosidase activity (Figure 10, panel A). Statistical analysis is presented in Fig. 10, panel B.

**Fig. 11:**
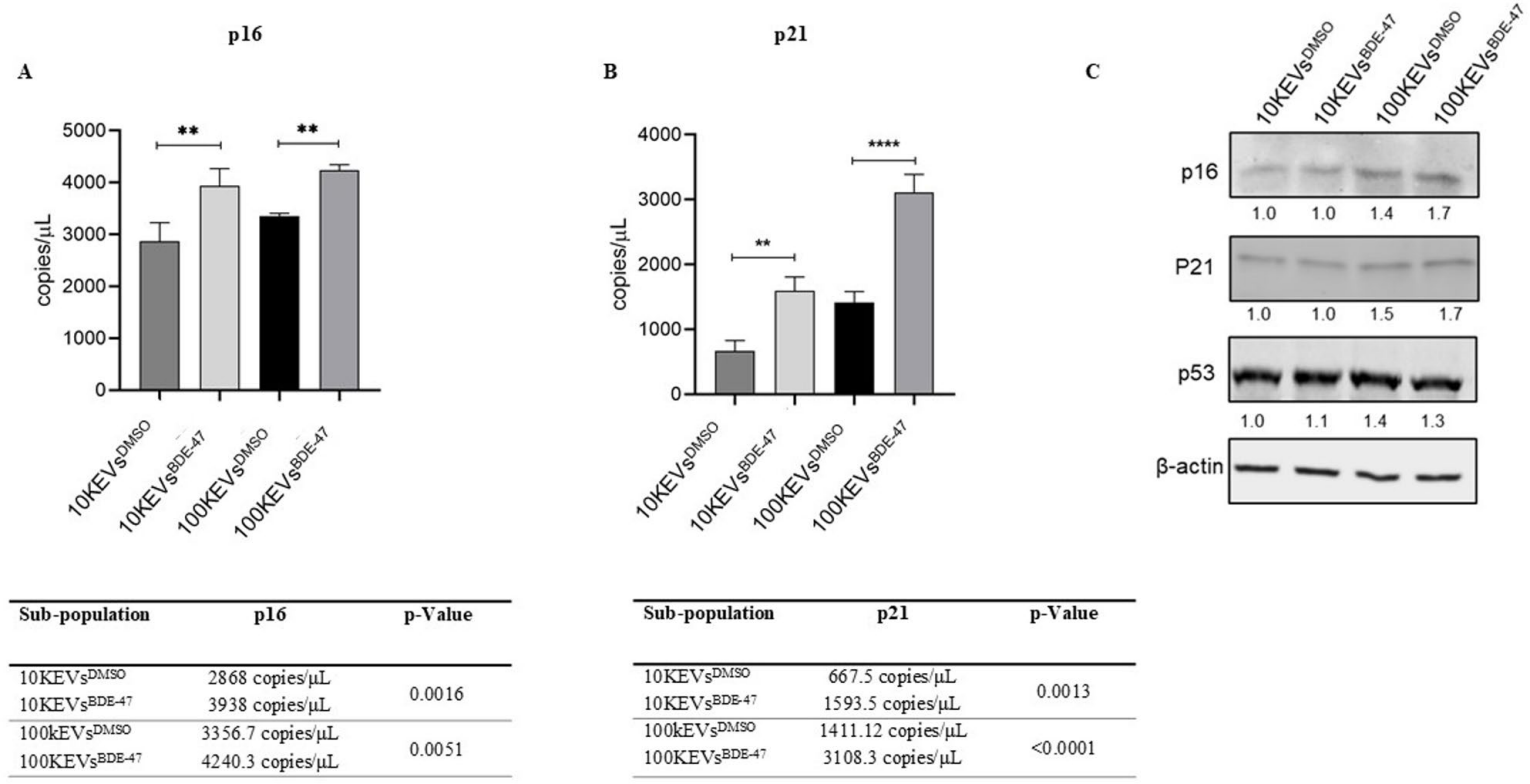
Evaluation of cyclin-dependent kinase inhibitor expression, p16 and p21 in LNCaP cells treated with 10KEVs^BDE−47/DMSO^ or 100KEVs^BDE−47/DMSO^ by digital PCR (**A** and **B**, **p < 0.005, ****p < 0.0001) and Western Blot assays (**C**) of p16, p21 and p53 in LNCaP cells treated with 10KEVs^BDE−47/DMSO^ or 100KEVs^BDE−47/DMSO^. NT indicates untreated cells

During cellular senescence, the cyclin-dependent kinase inhibitors p16 and p21 are typically upregulated. To investigate this, we assessed the expression of p16 and p21, encoded by the CDKN2A and CDKN1A genes, respectively. Digital PCR analysis (Fig. 11A) revealed a comparable increase in p16 mRNA copy number in LNCaP cells treated with either 10KEVs^BDE−47^ or 100KEVs^BDE−47^, relative to their respective controls. In contrast, p21 expression was significantly higher in cells treated with 100KEVs^BDE−47^ than in those treated with 10KEVs^BDE−47^ or 100KEVs^DMSO^, indicating a more robust activation of this senescence marker by 100KEVs following BDE-47 exposure (Fig. 11B). Finally, we assessed the expression levels of p16, p21 and p53 proteins in LNCaP cells treated with different EV enriched fractions. As shown by the Western blot analysis in Figure 11C, treatment with 10KEVs^BDE−47^ led to an upregulation of both p16 and p21 compared to the other conditions. Furthermore, the upregulation of p53, along with p21, indicates the involvement of the p53/p21 axis in the induction of cellular senescence [19]. This increased expression is consistent with the induction of a senescent phenotype and supports the role of 100KEVs^BDE−47^ in promoting cell cycle arrest.

## Conclusions

Recent research has highlighted a fundamental role of extracellular vesicles in coordinating physiological processes and transmitting stress signals under pathological conditions, reflecting dynamic responses to changes in the cellular microenvironment [20]. In toxicology, exposures to xenobiotics and environmental stressors can alter EV release and cargo composition, amplifying inflammatory responses and systemic effects through distal cell communication, a mechanism increasingly investigated for its potential as both a mechanistic mediator of toxicity and a biomarker of exposure and early effect [20, 21]. However, the analysis of this relationship remains complex and influenced by multiple variables, including the type of pollutant, duration and intensity of exposure, and the specific tissues or organs affected [22–25]. In this study, we performed an in vitro biophysical, molecular and functional characterization of extracellular vesicles secreted by THP-1 M(LPS) macrophages following exposure to the flame retardant BDE-47. The added value of this manuscript lies in the investigation of the heterogeneity of extracellular vesicles secreted by human macrophages upon pollutant exposure. In fact, EV studies are commonly performed on total EV preparations, without accounting for the intrinsic heterogeneity of different EV fractions. Our findings highlight the ability of environmental toxicants to reshape the molecular landscape of EVs’ and, in this way, modulate their downstream biological effects.

In our experimental set-up, differential ultracentrifugation enabled the isolation and biophysical characterization of EV fractions, referred to as 10KEVs^BDE−47^,10KEVs^/DMSO^, 100KEVs^BDE−47^ and 100KEVs^DMSO^. Immunoblot analysis of EV-associated markers was performed in accordance with the MISEV 2018 [11] and MISEV 2024 [12] guidelines and included the classical 100K EV markers (CD63 and CD81) as well as the 10K-associated marker calnexin. These analyses showed the expected distribution patterns in both control and BDE-47-treated samples, indicating that pollutant exposure did not alter the expression of the selected markers. The partial overlap in protein markers between the purified fractions highlights the inherent limitations of separation based solely on sedimentation velocity and reflects the intrinsic heterogeneity of extracellular vesicles [26, 27]. Accordingly, the two purified fractions should be considered enriched populations within a complex biological continuum rather than strictly discrete, mutually exclusive entities.

Surface glycans are central to extracellular vesicle–mediated communication, as they influence vesicle recognition, targeting, and uptake by recipient cells [28, 29]. More generally, carbohydrate-dependent interactions are key regulators of cell–cell communication, particularly in contexts such as host–pathogen interactions and the tumor microenvironment [30]. Lectins (carbohydrate-binding proteins) often serve as the receptors that regulate carbohydrate-mediated signaling, influencing the behavior of antigen-presenting cells and T-cells [31]. Glycoprofiling analysis on EVs enriched fractions revealed that BDE-47 stimulation altered the surface glycan composition of these vesicles. Using a panel of human C-type lectins, we observed that both 10KEVs^BDE−47/DMSO^ and 100KEVs^BDE−47/DMSO^ bind DC-SIGN and MGL, confirming the presence of N or O glycans on the EV’s surface and indicating an increased display of high mannose/fucosylated and GalNAc containing structures on the surface of BDE-47 treated cells with a specific enrichment of DC-SIGN–binding glycans in the 100KEVs^BDE−47^. In this context, we can hypothesise that the pollutant induced modulation of EV surface glycans could confer to macrophage derived EVs an altered capacity to engage DC-SIGN⁺/MGL⁺ myeloid cells in vivo, thereby impacting their uptake routes and regulatory signalling. Studies on glycosylated EVs, parasite-derived EVs and bacterial glycoconjugates [32] have shown that quantitatively similar changes in lectin recognition are sufficient to markedly alter EV uptake and immune activation. For examples, *S. mansoni schistosomula* EVs are internalized in a predominantly DC-SIGN-dependent manner, and DC-SIGN blockade almost completely abrogates uptake and associated cytokine responses [33]. Moreover, recent comprehensive overviews of EV glycosylation in cancer have emphasized that even moderate alterations in EV surface glycans can significantly affect EV biogenesis, uptake and organ tropism, as well as downstream metastatic and immune functions [34]. These data indicate that fold-changes of the magnitude we observe in DC-SIGN and MGL binding are within the range known to have functional consequences for EV-cell interactions. Furthermore, these findings may help explain our previously published observations, in which BDE-47–induced alterations in small EVs isolated from M(LPS) THP-1 macrophages were associated with functionally relevant immune modulation rather than passive bystander effects [6].

Beyond the modulation of glycan expression on surface’s EVs, BDE-47 also reprogrammed the miRNA cargo of both the 10KEVs^BDE−47/DMSO^ and the 100KEVs^BDE−47/DMSO^. Our microarray miRNA profiling revealed distinct signatures in each EV fraction, with no overlap between the miRNAs differentially expressed in the 10KEVs^BDE−47/DMSO^ versus 100KEVs^BDE−47/DMSO^, indicating a marked rewiring of miRNA sorting under toxicant exposure. A bioinformatic miRNA analyses advanced our understanding of BDE-47 impact on EV miRNA cargo showing a robust association between these BDE-47 modulated miRNAs and critical cellular processes related to senescence, cell cycle regulation, and oncogenic signalling pathways (for instance, NOTCH, AKT, ALK genes).

LNCaP cells are an androgen-responsive prostate cancer model that mount a robust senescence program under clinically relevant stresses (androgen deprivation, genotoxic therapy, irradiation, etc.). They consistently show canonical readouts (SA-β-gal positivity, growth arrest, and p21/p16 axis activation) and a measurable SASP (e.g., IL-6/IL-8), enabling mechanistic and biomarker studies. Together, these features make LNCaP an ideal system for probing proliferation–senescence dynamics [35]. Therefore, to assess the functional relevance of our molecular and bioinformatic findings, we first evaluated the uptake of the enriched EV fractions by LNCaP cells. We observed minimal internalization of 10KEVs^BDE−47/DMSO^ compared with the substantially higher uptake efficiency of the 100KEVs^BDE−47/DMSO^ fractions. The differential effects observed between the 10KEVs^BDE−47/DMSO^ and 100KEVs^BDE−47/DMSO^ fractions may reflect the functional heterogeneity of the investigated EV preparations whose uptake may depend on multiple factors, including vesicle size, surface composition, receptor interactions, etc. Such interactions could influence EV uptake pathways and downstream regulatory signaling. However, this axis was not directly investigated in our LNCaP-based functional assays, also due to the inhibitory effects of 100KEVs on cell proliferation, which made comparisons difficult. This represents a limitation of the present study and precludes us from establishing a role for DC-SIGN in the uptake of 100KEVs^BDE−47/DMSO^ uptake.

Instead, we further focused our study on the downstream effects of the 10KEVs^BDE−47/DMSO^ and 100KEVs^BDE−47/DMSO^ fractions on senescence-related pathways in LNCaP prostate cancer cells. Both 100KEVs derived from BDE-47- and DMSO-treated macrophages significantly reduced LNCaP cell proliferation compared with their corresponding 10KEV fractions, as demonstrated by BrdU incorporation and β-galactosidase senescence assays. These findings are consistent with the results of the uptake experiments. Moreover, 100KEVs^BDE−47^ showed a stronger anti-proliferative effect compared with 100KEVs^DMSO^, supporting the hypothesis that BDE-47 exposure enhances the biological activity of this EV fraction. Consistently, transcriptional analysis revealed a significant upregulation of the cell cycle inhibitors p16 and p21 in LNCaP cells treated with the EV-enriched fractions, further indicating the activation of growth-inhibitory pathways. Both p16 and p21 are key markers of cellular senescence and act by inhibiting cyclin-dependent kinases, leading to the inactivation of the retinoblastoma protein and arrest of the cell cycle in the G1 phase [36]. While p21 is typically activated in response to DNA damage and p53 signalling, p16 is more often linked to stress-induced or replicative senescence. Notably, in our setting, p21 upregulation was more pronounced in cells treated with the 100KEVs^BDE−47^ than in controls 100KEVs^DMSO^. This was confirmed by Western blot analysis, which revealed increased p21 expression specifically in 100KEVs^BDE−47^ treated LNCaP cells.

All together, these observations raise intriguing possibilities: the altered surface glycans and miRNA cargo of BDE-47-treated M(LPS) THP-1 macrophages may allow the release of EVs with enhanced bioactivity in modulating bystander cell behaviour. Such findings underscore the potential for BDE-47 to impact not only immune cells perturbating their response as previously shown [7, 8, 37] but also influencing heterologous cell dynamics via EVs. These results support the idea that EV-associated molecular signatures are not merely reflective of the cellular state of origin but can actively influence recipient cell responses, acting as mechanistic drivers of cellular reprogramming.

In addition, our findings highlight the importance of dissecting EV heterogeneity to fully understand how environmental insults can shape intercellular communication networks and contribute to pathophysiological processes, including immune dysregulation [37] and cell proliferation. The enhanced inhibitory effects of 100KEVs^BDE−47^ vs. 100KEVs^DMSO^ on LNCaP cell proliferation underscore the functional consequences of these molecular alterations. Indeed, given the stability and bioavailability of EVs in circulation, these vesicles may also serve as potential biomarkers for environmental exposure or as vehicles for systemic signal dissemination. Nevertheless, several limitations should be acknowledged. First of all, our analysis focused exclusively on the miRNA cargo and did not include functional validation of individual miRNAs or other EV cargo components. In addition, the molecular mechanisms underlying the BDE-47–induced alterations in EV fractions, as well as the pathways governing their cellular uptake and downstream mechanisms on LNCaP were not investigated. Further research is needed to elucidate how BDE-47 affects the molecular machinery involved in miRNA sorting and the enzymatic pathways regulating glycosylation. Such insights would significantly advance our understanding of toxicant-induced vesicle reprogramming, which was not directly addressed in the present study. Finally, in vivo studies addressing the biodistribution, immune interactions, and systemic effects of EVs derived from BDE-47–treated macrophages would provide important translational insight.

In conclusion, this study demonstrates that exposure to the flame-retardant BDE-47 selectively reshapes the molecular and functional landscape of macrophage-derived EVs. While their overall biophysical features remain preserved, significant alterations in glycosylation patterns and miRNA cargo have been described that could translate into modified effects on recipient cells. These findings open new perspectives on the role of EVs as mediators of environmental toxicity and support their potential utility as biomarkers of pollutant-induced health risk.

## Supplementary Information

Supplementary Material 1.

Supplementary Material 2.

## Data Availability

All data supporting the findings of this study are available within the paper and Supplementary Information.

## Abbreviations

BrdU: Bromodeoxyuridine
BSA: Bovine Serum Albumin
BDE: 47-2,2’,4,4’-tetrabromodiphenyl ether
DC SIGN: Dendritic Cell-Specific Intercellular adhesion molecule-3-Grabbing Non-integrin
DTT: Dithiothreitol
DLS: Dynamic light scattering
DMSO: Dimethyl sulfoxide
dUC: differential ultracentrifugation
Eno: 1-Enolase-1
ESCRT: Endosomal Sorting Complex Required for Transport
EVs: Extracellular vesicles
FBS: Fetal Bovine Serum
lEVs: Large Extracellular vesicles
LPS: Lipopolysaccharides
MGL: Macrophage Galactose-type Lectin
MR: Mannose receptor
MVEs: Multivesicular endosomes
MVs: Microvesicles
NTA: Nanoparticle Tracking Analysis (NTA)
OD: optical density
PBDEs: Polybrominated diphenyl ether
PBS: Phosphate buffered saline
PDI: Polydispersity index
PMA: Phorbol 12-myristate-13-acetate
PVDF: Polyvinylidene fluoride
RPMI 1640: Roswell Park Memorial Institute 1640
SASP: Senescence-Associated Secretory Phenotype
SDS: Sodium dodecyl sulphate
sEVs: small Extracellular vesicles
TMB − 3,3′,5,5′: tetramethylbenzidine
TSM: Tris(hydroxymethyl)aminomethane
