# Supplementary figures and images for: Environmental pollutant BDE-47 alters glycan and microRNA signatures of macrophage-derived extracellular vesicles and modulates senescence signaling
Source: Cell Commun Signal. 2026 May 6;24:377. doi: 10.1186/s12964-026-02903-2 (PMC13312629; doi:10.1186/s12964-026-02903-2)

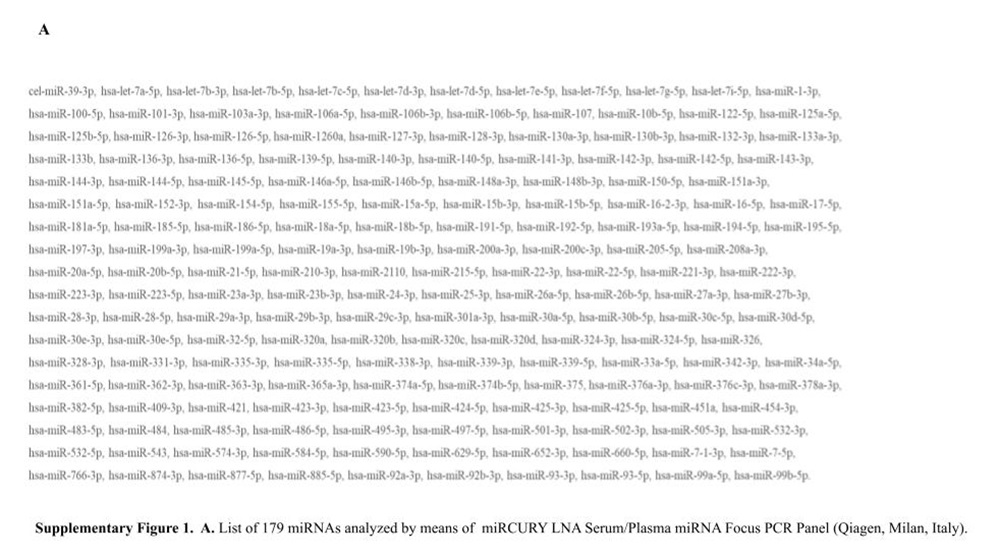

Supplement: Supplementary file 1 — Supplementary Material 1. [file 12964_2026_2903_MOESM1_ESM.jpg]

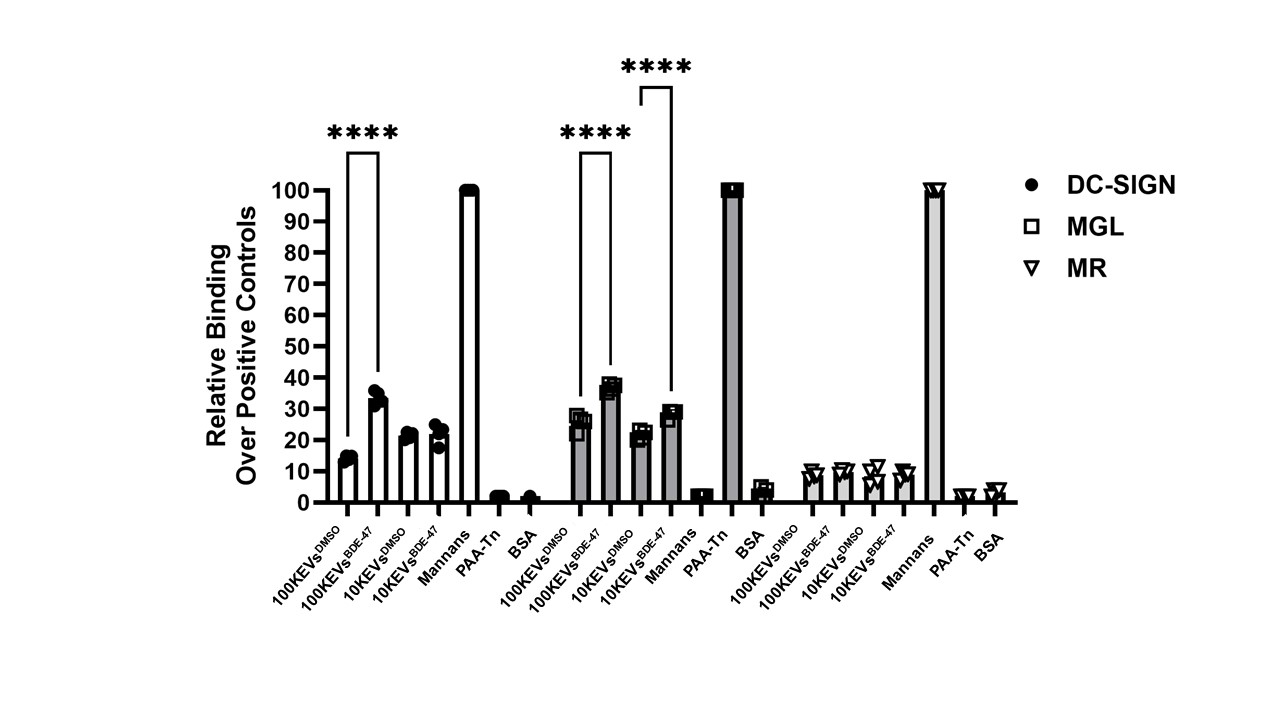

Supplement: Supplementary file 2 — Supplementary Material 2. [file 12964_2026_2903_MOESM2_ESM.jpg]
